# Metacarpophalangeal joint reconstruction using a costal osteochondral graft: A case report

**DOI:** 10.1097/MD.0000000000037868

**Published:** 2024-04-19

**Authors:** Chien-Liang Ho, I-Ying Lee, Hsiu-Yun Hsu, Li-Chieh Kuo, Jing-Jing Fang

**Affiliations:** a Division of Plastic and Reconstructive Surgery, Department of Surgery, National Cheng Kung University, Tainan, Taiwan; b Department of Occupational Therapy, College of Medicine, National Cheng Kung University, Tainan, Taiwan; c Department of Physical Medicine and Rehabilitation, National Cheng Kung University Hospital, College of Medicine, National Cheng Kung University, Tainan, Taiwan; d Medical Device Innovation Center, National Cheng Kung University, Tainan, Taiwan; e Department of Mechanical Engineering, National Cheng Kung University, Tainan, Taiwan.

**Keywords:** case report, costal osteochondral graft, giant cell tumor, joint reconstruction, three-dimensional printing

## Abstract

**Rationale:**

The conventional treatment of giant cell tumors is intralesional curettage with local adjuvant therapy. Because hand tumors have a high local recurrence, the primary goal for treating tumors of the hand is to eradicate the lesion.

**Patient concerns:**

To preserve the metacarpophalangeal (MCP) joint function as well as avoid further recurrence after surgery.

**Diagnoses:**

The giant cell tumor invades the patient’s MCP joint in an index proximal phalanx.

**Interventions:**

Using computer-aided design and three-dimensional printing techniques, we reformed the original shapes of the MCP joint and its peripheral bone to replica models. The surgeon then performed an en bloc resection and proximal phalanx with MCP joint reconstruction by fabricating the patient’s costal osteochondral graft during the operation.

**Outcomes:**

After 6 months of rehabilitation, the patient’s finger functions could pinch and grasp objects naturally. At the 1-year follow-up, the range of motion of the MCP, proximal interphalangeal, and distal interphalangeal joints improved from flexion of 35° to 60°, 75° to 85°, and 60° to 80°, respectively. The hand function achieved the mean performance of non-preferred hands for young females at the postoperative 3-year follow-up.

**Lessons:**

The customized prototyping technique has the potential to replica the original patient’s bony graft to reach the goal of minimizing the defects at the donor site and maximizing the function of the reconstructed MCP joint.

## 1. Introduction

A giant cell tumor (GCT) of the hand bone is approximately 2% to 5% in skeletally mature patients.^[1,2]^ The conventional treatment is intralesional curettage with local adjuvant therapy.^[3]^ According to the high local recurrence of hand tumors, the primary goal for treating tumors of the hand is to eradicate the lesion.^[1]^ However, to ensure the comprehensive utility of human hands in daily activities, surgeries for hand tumors significantly impact functional performance and quality of life for these patients. The dilemma or challenge of balancing preserving function, improving cosmetic outcomes, and decreasing the local recurrence rate should be a critical issue when dealing with this medical condition.^[3]^ Recent advancements in medical imagery and computer technology have been reported to be powerful tools to revolutionize performance in complicated hand surgeries.^[4]^ The human hand is characterized as a complex, intricate anatomic structure. Therefore, using a computer-aided design (CAD) fabricated by a three-dimensional printing (3DP) technique^[5]^ is a feasible approach in preoperative planning and simulation for hand surgeries.

## 2. Case report

**Figure 1.**
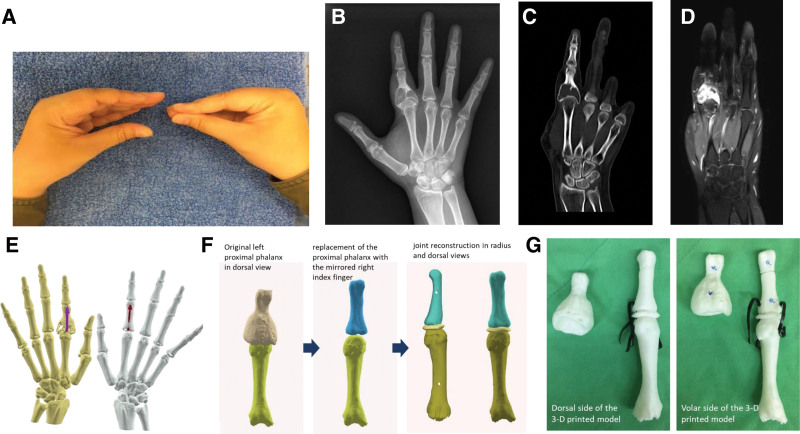
Preoperative images: (A) the limited ROM of the left MCP joint before surgery; (B) X-ray shows a radiolucent lesion with a thinning cortex; (C) CT reveals an intact bony structure but a thinning cortex and no joint space involvement; (D) MRI-T2 weight indicates no soft tissue involvement; (E) 3D lesions compared with a typical presentation; (F) procedures for reconstruction; and (G) Dorsal and volar views of the 3D printed model. 3D = three-dimensional, CT = computed tomography, MCP = metacarpophalangeal, MRI = magnetic resonance imaging, ROM = range of motion.

The National Cheng Kung University Hospital Institutional Review Board approved the case report, numbered B-EC-112-024. This 20-year-old female had undergone an operation in a local clinic 4 years before this intervention and was diagnosed with lipoma. However, the tumor enlarged gradually over the years. Due to the progression of symptoms (Fig. 1A), the patient visited the plastic surgery outpatient clinic for a surgical consult. Radiographs revealed a 2.6 × 2.3 cm translucent lesion on the proximal half of the proximal phalanx of the left index finger. Computed tomography and magnetic resonance imaging were scheduled for further characterizing and diagnosing a Grade II giant cell tumor (Fig. 1B–D).

**Figure 2.**
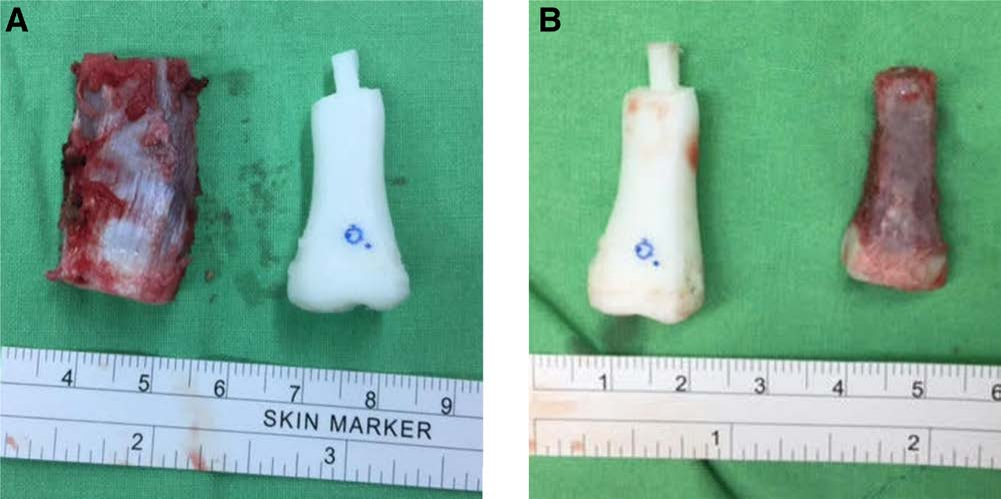
(A) The costal osteochondral graft and the target model. (B) Graft after fabrication.

After discussion, an en bloc surgery for the patient’s left index finger was planned to replace the proximal phalanx and reconstruct the metacarpophalangeal (MCP) joint. We first performed an osteotomy and reconstruction processes simulated by computer-aided software to reconstruct her proximal phalanx and MCP joint, then fabricated the physical model using a 3DP technique (Fig. 1E). A mirror image of the proximal phalanx of the right index finger was used to reproduce the target osteochondral graft for the lesion site (Fig. 1F). The MCP joint was recreated using Boolean operations. Specific and custom three-dimensional hand anatomical models were separately produced and then assembled with elastic rubber bands to simulate the MCP joint’s collateral ligaments to establish the joint’s limited movement (Fig. 1G). During the operation, the costal osteochondral graft was fabricated to mimic the shape of the target models (Fig. 2). We preserved the attachment of the lumbrical muscles and the radial site collateral ligament with a piece of the original bone cortex. We fixed the graft on the residual proximal phalanx with Kirschner wires. An X-ray immediately following the operation showed no wire fractures or bony collapse. A dynamic traction splint was applied 1 week after surgery to preserve the MCP joint space and allow early finger motion. Occupational therapy was started 4 weeks postoperatively to improve range of motion (ROM), build strength, and enhance hand functions. The traction splint was removed 2 months after surgery. A finger gutter splint was prescribed to prevent ulnar deviation of the proximal interphalangeal joint. Compared with her baseline condition shown in Table 1, the ROM of the MCP joint improved from flexion of 35° to 60°. The ROM of the proximal interphalangeal joint improved from flexion of 75° to 85°. The ROM of the distal interphalangeal joint improved from flexion of 60° to 80° at the 1-year follow-up. The outcomes were well maintained at the postoperative 3-year follow-up. Grasp and pinch strength were maintained and improved after rehabilitation. The hand function was evaluated using the Purdue Pegboard test and achieved the mean performance of non-preferred hands for young females^[6]^ at the postoperative 3-year follow-up. Hand magnetic resonance imaging at the 1-year follow-up showed no local recurrence.

**Table 1:** Three-year follow-up of changes in the range of motion of hand joints, hand strength, and function.

	Pre-op: PROM/AROM	Post-op 1M AROM	Post-op 6M PROM/AROM	Post-op 1Y PROM/AROM	Post-op 2Y PROM/AROM	Post-op 3Y PROM/AROM
MCPJ	0–40°/0–35°	0–40°	0–65°/0–60°	0–60°/0–60°	0–70°/0–65°	0–75°/0–65°
PIPJ	−10 to 75°/−10 to 75°	0–35°	0–90°/10–85°	0–90°/0–85°	0–95°/0–85°	0–95/0–90°
DIPJ	0–60°/0–60°	0–15°	0–85°/0–80°	0–90°/0–80°	0–90°/0–75°	0–80°/0–75°

			Post-op 6M Affected hand/non-affected hand	Post-op 1Y Affected hand/non-affected hand	Post-op 2Y Affected hand/non-affected hand	Post-op 3Y Affected hand/non-affected hand
Grasp power (kg)			28/36	31.3/35	25.3/35.6	28.0/35.6
Pinch power (kg)			1.5/3.5	2.5/4.3	2/3.5	2.5/4.1
Lateral pinch power (kg)			5/8	4.8/7.7	6/7.5	6.1/7.7
Purdue Pegboard test (Pin insertion subtest) (number of pins)			15.5/17.7	16.0/17.7	17.3/19.7	18.0/19.0

AROM = anterior range of motion, DIPJ = distal interphalangeal joint, MCPJ = metacarpophalangeal joint, PIPJ = proximal interphalangeal joint, Post-op = postoperative, Pre-op = preoperative, PROM = posterior range of motion.

## 3. Discussion

There was no definite consensus about treatment options for the relatively rare incidence of a finger bone tumor, and very few reconstructive operations have been reported. Three different approaches to resection of a tumor, including intralesional curettage, ray amputation, and en bloc surgery, as well as reconstruction of the MCP joint and proximal phalanx and the MCP joint, including both proximal phalanx and metacarpal bone, have been considered for treating a GCT. Intralesional curettage was not suitable for this patient because very little cortex was left after the operation, and there was a high risk of structural collapse and local recurrence.^[1,2]^ Ray amputation allows an en bloc surgery and relatively good dexterity compared with finger amputation.^[2,7]^ Also, an en bloc surgery to remove the MCP joint, including the base of the proximal phalanx and the head of the metacarpal bone, with total replacement, can provide a better cosmetic outcome and functional preservation. However, risks of graft failure and dominant donor site defects have been reported.^[2]^ The en bloc surgery with removal of only the base of the proximal phalanx following MCP joint reconstruction with the costal osteochondral graft had the advantage of total replacement of the MCP joint but with fewer donor site defects. However, it is challenging to reconstruct and maintain the joint space with an unknown graft failure rate. To consider the psychological challenges for a young lady, the en bloc surgery only removing the proximal phalanx bone following reconstruction of the MCP joint was carried out to preserve the appearance and function of the hand and minimize donor site defects.

We performed a systematic review of a total of 6 case reports and 1 case series, which led to a total of 15 cases with GCT,^[8–12]^ aneurysm bone cysts,^[12]^ atypical cartilaginous lesion,^[12]^ or traumas^[13,14]^ undergoing surgery involving 1 bony resection with MCP joint reconstruction (Appendix 1, http://links.lww.com/MD/M229). For patients with a GCT, the en bloc excision of the affected bones was the option for 5 patients,^[11,12]^ intralesional curettage was used for 2 patients,^[8,10]^ and radical resection was used for 1 patient^[9]^ as the primary treatment. Tumor recurrence was not reported for 8 patients during the 6 to 70-month follow-up period. As for the reconstruction of joint defects due to GCT revision surgery, 4 patients received MCP joint reconstruction with a nonvascularized metatarsal autograft transfer,^[12]^ 1 underwent a vascularized metatarsal autograft transfer,^[8]^ 1 patient received silicone arthroplasty,^[11]^ and 2 received an osteochondral autograft.^[9,10]^ For all 15 patients undergoing MCP joint reconstruction, the ROM of the MCP joint was at least 60° and most preserved nearly normal functions except for 1 patient receiving surgical arthrodesis of the joint following a pin tract infection.^[12]^ The ROM outcome for the MCP joint reconstruction in all the reviewed cases was similar to that reported in the case reported herein. However, resorption of the bone graft in the hand has been reported to be a late complication when using a nonvascularized metatarsal shaft,^[15]^ and an implant fracture has been considered a disadvantage when using silicone arthroplasty^[16]^ for reconstruction. In our review, only 1 study revealed the hand strength outcome after surgery for GCT of the hand bones, even though it has been considered an important outcome parameter for hand surgeries.^[8]^ Our case report assessed the pinch and grip strength using a Jamar gauge and compared the nearly average results with the contralateral hand. At her 1-year follow-up, the grip strength in her left hand was 89.4% of that of the right hand, in addition to excellent finger motion preservation. The results were maintained, and no evidence of tumor recurrence was found at the 3-year follow-up. Thus, using a CAD with a 3DP technique to simulate MCP joint reconstruction provided a potential strategy leading to superior function compared to alternative practices, such as metatarsal transfer and silicone arthroplasty.

**Figure 3.**
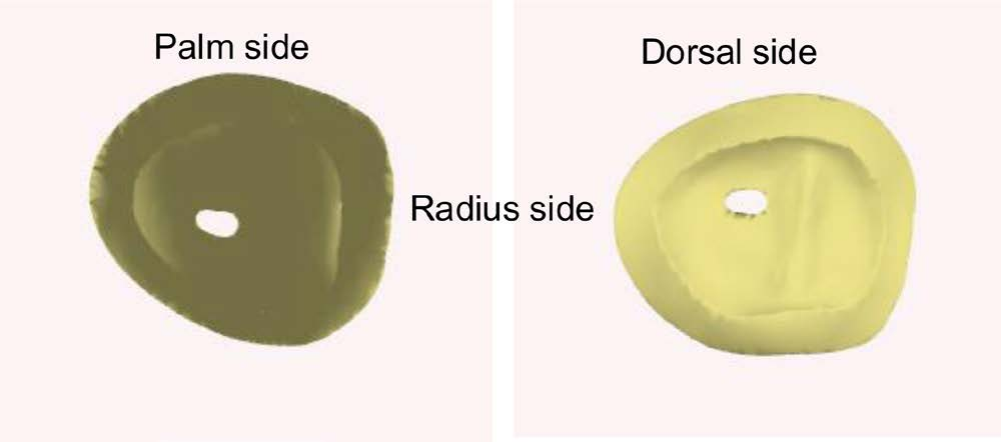
Metacarpophalangeal joint of the left second metacarpal.

One of the challenges of the MCP joint reconstruction with the costal osteochondral graft was the modification of the bone graft’s shape and orientation. CAD and 3DP techniques were used to simulate and fabricate the target models for the surgery. The defect structure was reconstructed by mirroring the normal contralateral side of the 2nd metacarpal head, as shown in Figure 3. Using these personalized models, the residual distal proximal phalanx could be precisely preserved, and a bone graft with an articular surface that fits the original metacarpal head could then be constructed. This accurately reproduced the condition of the patient’s finger during the operation. The difficulty and limitation of sculpting during surgery is that a costal osteochondral graft should be used. To sculpt a proximal phalangeal shaft, part of the surrounding cortical bone must be sacrificed, which will affect the structure of the graft and the stability of the surrounding bone fixation. Tissue engineering for osteochondral defects with MCP joint involvement is still challenging and has failed to be achieved. In conclusion, a CAD with 3DP technology has the potential to customize the bony graft to reach the goal of minimization of the defects at the donor site and maximization of the function of the reconstructed MCP joint.

## Author contributions

**Conceptualization:** Chien-Liang Ho, Jing-Jing Fang.

**Data curation:** I-Ying Lee, Hsiu-Yun Hsu, Li-Chieh Kuo.

**Methodology:** Chien-Liang Ho, Jing-Jing Fang.

**Software:** Jing-Jing Fang.

**Writing – original draft:** Chien-Liang Ho, I-Ying Lee.

**Writing – review & editing:** Jing-Jing Fang.

## Supplementary Material


