# Supplementary material for: Metacarpophalangeal joint reconstruction using a costal osteochondral graft: A case report
Source: Medicine (Baltimore). 2024 Apr 19;103(16):e37868. doi: 10.1097/MD.0000000000037868 (PMC11029923; doi:10.1097/MD.0000000000037868)
Supplement: Supplementary file 1 [file medi-103-e37868-s001.docx]

**Appendix 1.** Individual patient characteristics, treatments, and outcomes of the review case study and case series

| **Study** | **Patient/Gender /Age (years)** | **Site** | | **Primary Treatment/ Reconstruction** | **Follow-up**  **(months)** | **Outcomes (ROM)** | | |
| --- | --- | --- | --- | --- | --- | --- | --- | --- |
|  |  | **Left** | **Right** |  |  | **MCPJ** | **PIPJ** | **DIPJ** |
| Menonet et al. (1983) ^10^ | 1/M/22 | IF GCT  MC+MCPJ |  | Resected of the distal 3/4 of the second MC together with portions of the intrinsic muscles / The 2^nd^ metatarsal was transplanted with the cuff of the intrinsic m. with radial collateral ligament anchoring. | 27 | 0º~80º | 0º ~100º | 0º~65º |
| Kakinoki et al. (2008) ^15^ | 3/M/56 | LF trauma  PP+MCPJ |  | Removal of the PP and proximal articular surface of the MP / A partial fifth metacarpus, which was vascularized by the fourth dorsal metacarpal vessels, was transplanted. | 12 | 0º~60º | 0º~60º | 0º~10º |
| Sahin et al. (2007) ^16^ | 2/M/38 | RF trauma  PP+MCPJ |  | Bone graft with artificial artery insertion for bone defects. | 6 | 0º~60º | 0º~60º | 0º~10º |
| Neil et al. (2012) ^11^ | 4/F/66 |  | RF GCT MC+MCPJ | Radical resection of the MC and the proximal surface of the PP/ MC and MCPJ reconstructed with a fibular osteocutaneous free flap and silicone arthroplasty, respectively. | 54 | 15º~85º | 10º~90º | 0º~50º |
| Spiro et al. (2012) ^12^ | 5/F/31 |  | IF GCT  PP+MCPJ | Curettage of PP/ Reconstruction with an osteochondral autograft from the ipsilateral distal femoral condyle | 24 | 0º~80º | - | - |
| Ansari et al. (2014) ^13^ | 6/F/32 | IF GCT  PP+MCPJ |  | En bloc resection of the PP/ fibular autograft and silicone implant arthroplasty to reconstruct the PP and MCPJ. | 18 | NA from the manuscript report | - | - |
| Samuel et al. (2016) ^14^ | 7/M/29 |  | LF ABC  MC+MCPJ | En bloc excision of the MC and MC heads / nonvascularized metatarsal shaft and head harvested from the foot to replace the MC and MCPJ defect | 12 | 0º~90º | - | - |
|  | 8/F/14 |  | MF ABC  MC+MCPJ |  | 104 | 0º~90º | - | - |
|  | 9/F/15 |  | MF ABC  MC+MCPJ |  | 26 | 0º~80º | - | - |
|  | 10/M/31 | IF GCT  MC+MCPJ |  |  | 28 | 0º~80º | - | - |
|  | 11/F/13 |  | MF GCT  MC+MCPJ |  | 70 | 0º~85º | - | - |
|  | 12/M/23 | MF ABC  MC+MCPJ |  |  | 30 | 0º~90º | - | - |
|  | 13/M/14 |  | Thumb GCT  MC+MCPJ |  | 52 | Fused | - | - |
|  | 14/F/26 |  | RF GCT  MC+MCPJ |  | 35 | 0º~80º | - | - |
|  | 15/F/45 |  | LF ACL  MC+MCPJ |  | 27 | 0º~85º | - | - |

IF: index finger, MF: middle finger, RF: ring finger, LF: little finger, MC: metacarpal, PP: proximal phalange, MCPJ: metacarpophalangeal joint, PIPJ: proximal interphalangeal joint, DIPJ: distal interphalangeal joint, ROM: range of motion, ABC: aneurysmal bone cyst, ACL: atypical cartilaginous lesion, GCT: giant cell tumor, NA: not available.
